# Point-of-care human milk testing for maternal secretor status

**DOI:** 10.1007/s00216-021-03697-7

**Published:** 2021-11-05

**Authors:** Saeromi Chung, Lars Bode, Drew A. Hall

**Affiliations:** 1 Department of Electrical and Computer Engineering, University of California San Diego, La Jolla, CA 92093 USA; 2 Department of Pediatrics, University of California San Diego, La Jolla, CA 92093 USA; 3 Mother-Milk-Infant Center of Research Excellence (MOMI CORE), University of California San Diego, La Jolla, CA 92093 USA; 4 Department of Bioengineering, University of California San Diego, La Jolla, CA 92093 USA

**Keywords:** Human milk oligosaccharides, 2′-Fucosyllactose, Impedance sensor, Milk analyzer, Human milk

## Abstract

We present an electrochemical impedimetric-based biosensor for monitoring the variation in human milk oligosaccharide (HMO) composition. 2′-Fucosyllactose (2’FL) is an HMO associated with infant growth, cognitive development, and protection from infectious diarrhea, one of the major causes of infant death worldwide. Due to genetic variation, the milk of some women (non-secretors) contains no or very little 2′FL with potential implications for infant health and development. However, there is currently no technology to analyze the presence and concentration of HMOs in human milk at the point-of-care (POC). The lack of such technology represents a major impediment to advancing human milk research and improving maternal-infant health. Towards this unmet need, we report an impedimetric assay for HMOs with an α-1,2 linkage, the most abundant of which is 2′FL. The sensor uses a lectin for affinity, specifically *Ulex europaeus* *﻿agglutinin* *I* (UEA), with electrochemical readout. In spiked studies, the sensor exhibited a high degree of linearity (*R*^2^ = 0.991) over 0.5 to 3.0 μM with a 330-nM detection limit. The sensor performance was clinically validated using banked human milk samples and correctly identified all secretor vs. non-secretor samples. Furthermore, despite the short 35-min assay time and low sample volume (25 μL), the assay was highly correlated with HPLC measurements. This bedside human milk testing assay enables POC, “sample-to-answer” quantitative HMO measurement, and will be a valuable tool to assess milk composition.

**Graphical abstract:**

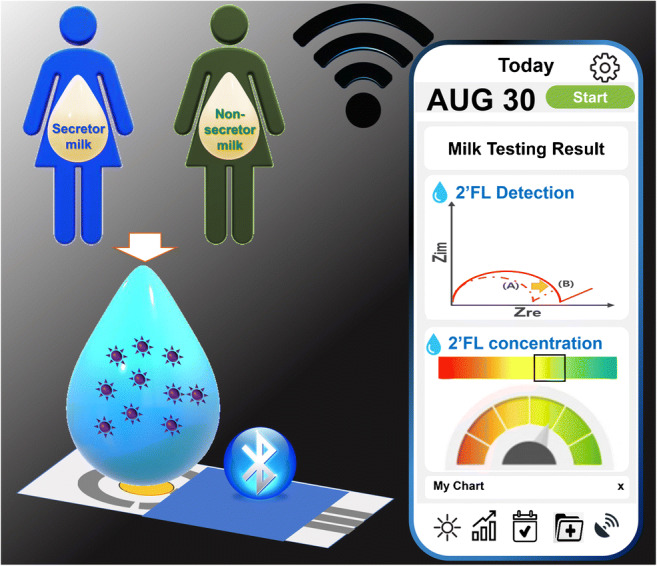

**Supplementary Information:**

The online version contains supplementary material available at 10.1007/s00216-021-03697-7.

## Introduction

Human milk is widely accepted as the best source of nutrients for healthy term newborns [1, 2], and the composition is strongly correlated with maternal-infant health and developmental outcomes [3, 4]. Studies have found that through universal breastfeeding, the death of 823,000 children and 20,000 mothers annually could be averted along with an economic savings of $300 billion [5]. While the benefits of human milk and breastfeeding are numerous, human milk composition varies between women and is dynamic over the course of lactation. Human milk oligosaccharides (HMOs) are a group of ~150 complex glycans (sugars) and, at 5–10 g/L, represent the third most abundant solid component of human milk after lactose and lipids [6–9]. Several HMOs, like 2′-fucosyllactose (2′FL), are present in high concentrations in most women (secretors) but are nearly absent in the milk of other women (non-secretors) [10]. Differences in maternal HMO composition have been linked to several short- and long-term infant health and disease outcomes. For example, the near absence of 2′FL in the milk of non-secretor women is associated with a higher infant risk of infectious diarrhea [3, 4], which remains one of the leading causes of death in infants and children under the age of 5 worldwide [11, 12]. Higher 2′FL concentration has also been associated with improved infant growth [13, 14], improved memory and learning in rodent models, and improved infant cognitive development at 24 months in human cohorts [15, 16]. While these and other studies highlight the importance of HMOs in human milk, no point-of-care (POC) technology is currently available to quantify the amount of 2′FL in human milk.

HMOs are unconjugated sugars (*i.e.*, they are not covalently bound to proteins or lipids) but instead carry lactose at the reducing end. Lactose can be elongated by adding one or more disaccharides and modified by adding fucose and/or sialic acid with different linkages [9]. 2′FL, lactose fucosylated with an α-1, 2 linkage at galactose, is the most abundant HMO in the milk of secretors [16]. Lectins are carbohydrate-binding proteins that recognize specific glycan structures [17, 18] and thus can be used as affinity reagents. They have been used as recognition molecules to capture glycan targets (*i.e.*, lectin-affinity chromatography, lectin-based biosensors, etc.) [19]. Specifically, *Ulex europaeus agglutinin I* (UEA), which has a binding site for α-1,2 linkages [20], is a natural binding partner for 2′FL (*K*_d_ = 327 nM) [21]. We hypothesized that UEA could be used as an affinity molecule to selectively capture 2′FL out of the higher abundance HMOs present in human milk.

The lack of technology to analyze human milk bioactives at the POC represents a major impediment to advancing human milk research and improving maternal-infant health. Over the past few decades, analytical methods to detect and quantify 2′FL have been developed. Early research focused on separating 2′FL and other HMOs using chromatographic techniques [22–24]. Unfortunately, chromatography is still a costly and time-consuming process requiring large amounts of organic solvent and sample [25]. To overcome these disadvantages, high-performance liquid chromatography (HPLC) was coupled with other techniques (*e.g.*, mass spectrometry (MS) [26], nuclear magnetic resonance (NMR) [27], and electrochemical techniques [28]) to detect HMOs. These approaches enhanced the sensitivity and selectivity, which allowed for the characterization of fucosylated oligosaccharides; however, they are limited to laboratory-based tests and not amenable to POC testing [29]. Currently, a significant obstacle in developing a POC testing device is that HMO analytical detection methods require burdensome sample pre-treatment, expensive instrumentation, and trained professionals to run the assays. Some macronutrients like total protein, total lipids, and lactose can be measured by “milk analyzers” at the bedside; however, the analysis of many other essential human milk components currently require specialized, expensive, and time-consuming technology that is available in only a handful of research laboratories around the world. Thus, despite the importance of HMOs to infant health, there is currently no sensor platform capable of detecting 2′FL without sending human milk samples to one of the very few research laboratories.

**Fig. 1:**
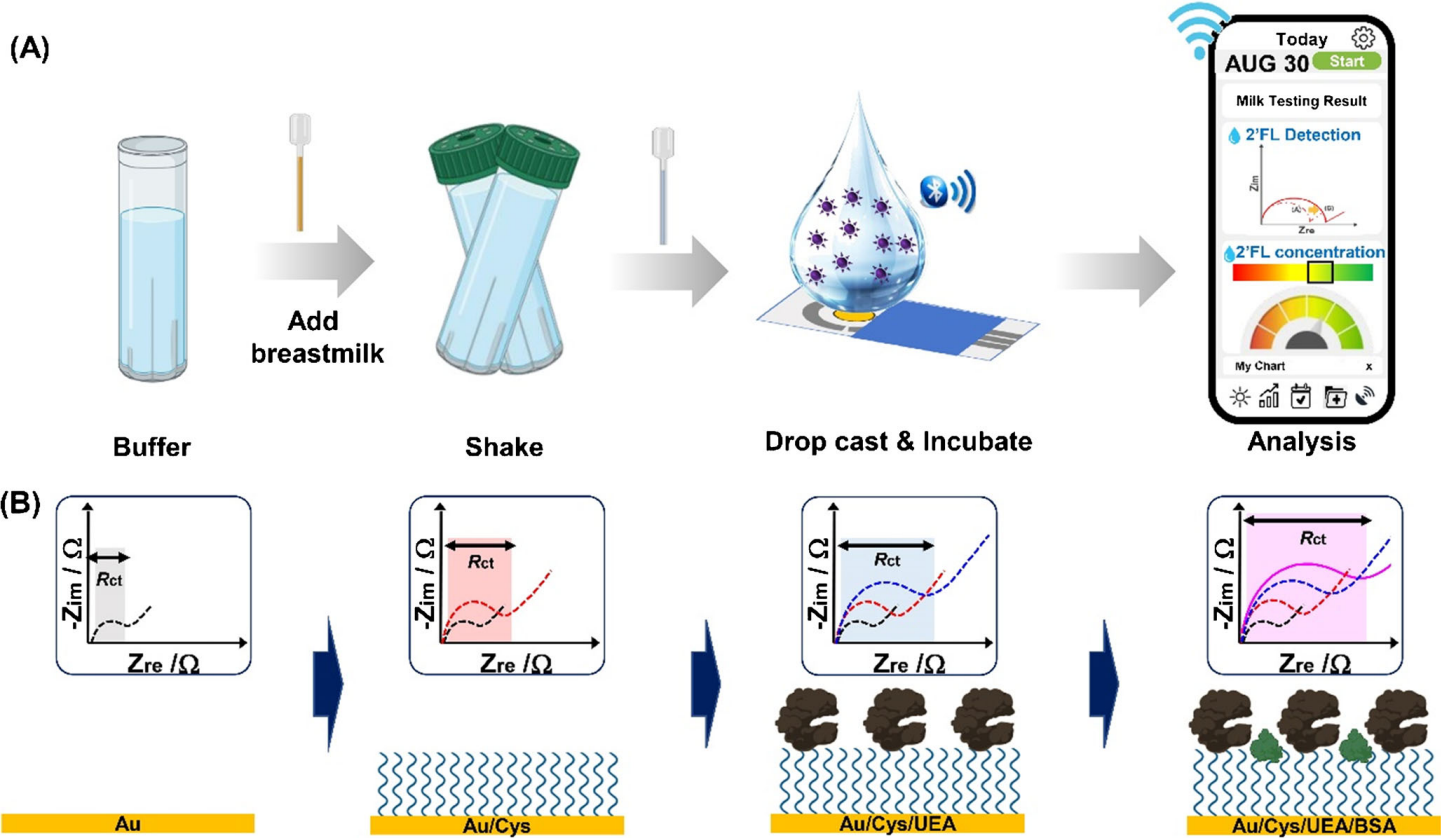
Overview of proposed human milk assay. (A) Workflow of human milk 2′FL bedside testing. (B) Illustrations of 2′FL sensor fabrication (inset: Nyquist plot of each layer shows the change in charge transfer resistance, *R*_ct_)

This study reports a rapid, label-free human milk screening assay to detect and quantify 2′FL as a biomarker to differentiate secretor vs. non-secretor status, as shown in Fig. 1. We used a lectin (UEA) covalently bound to an amine-functionalized gold electrode as the capture molecule. Upon binding with the cognate glycan (2′FL), the electrode impedance is perturbed and read out using electrochemical impedance spectroscopy (EIS). This electrochemical approach overcomes the disadvantages of the previously reported milk analysis methods. The assay requires minimal sample volume (25 μL), enabling POC, “sample-to-answer” quantitative measurement of HMOs. In summary, this assay allows a handheld POC solution for mothers and human milk studies with simple, rapid, and accurate HMO quantification.

## Materials and methods

### Reagents and instruments

2′-Fucosyllactose (2′FL; #SMB00933), phosphate-buffered saline (PBS; #P5493), glutaraldehyde (#G5882), sulfuric acid (#339741), cystamine dihydrochloride (#30050 sigma), human serum albumin (HSA; #A9511-100 mg), and ammonium hydroxide (#09859) were purchased from Sigma-Aldrich (USA). Disialyllacto-N-tetraose (DSLNT; #OD00172) and *Ulex Europaeus agglutinin I *(UEA; #L-1060) were purchased from Carbosynth (UK) and Vector Laboratories (USA), respectively. Bovine serum albumin (BSA; #37525), glucose (#A16828), Koptec pure ethanol (200 proof), and hydrogen peroxide (#HX0635-3) were obtained from Thermo Fisher Scientific (USA), Alfa Aesar (USA), Decon Labs (USA), and EMD Millipore, respectively. Bio-banked human milk samples were provided from the Mother-Milk-Infant Center of Research Excellence (MOMI CORE) at UCSD.

Voltammetry and electrochemical impedance spectroscopy measurements were performed using a benchtop potentiostat (CH Instruments, 750E), a portable multi-potentiostat (PalmSens, Palmsens4), and a custom-built, handheld potentiostat. A 3-electrode setup was used for characterization experiments with Au, Ag/AgCl, and Pt wire (CH Instruments, #CHI115) as the working (WE), reference (RE), and counter electrodes (CE), respectively. The 5-mm-diameter WE was formed with a custom fixture over a gold-sputtered glass slide (100 nm thick 99.999% pure Au film on a 5-nm Cr adhesion layer).

### Biosensor fabrication

Fabrication of UEA-based sensors is a stepwise process. First, Au-coated slides were chemically cleaned using piranha solution (a 3:1 mixture of H_2_SO_4_ and H_2_O_2_), then thoroughly washed with distilled water [30]. Next, the WE was electrochemically cleaned by sweeping the potential from 0 to 1.4 V (vs. Ag/AgCl) in 0.5 M H_2_SO_4_ [31]. After washing with distilled water, the Au surface was dried with compressed air. The surface was amine-functionalized by incubating the Au electrodes with 50 μL of 250 mM cystamine dihydrochloride (Cys) solution in a 1:4 mixture of NH_4_OH and ethanol overnight at room temperature. After washing with ethanol, 50 μL of 25% glutaraldehyde (amine-reactive bifunctional cross-linking reagent) was drop-cast on the electrodes and incubated for 1 h. Electrodes were then washed with distilled water 3×. Immediately afterward, 50 μL of 100 μg/mL UEA was incubated with the substrate for 2 h at room temperature and 8 h at 4 °C. Lastly, the electrodes were washed with PBS and incubated with 1% BSA for 30 min. After washing the electrodes, the electrode responses were compared before quantifying 2′FL to select those having a similar response.

### Sensor characterization

The stepwise modification of the electrodes was characterized using cyclic voltammetry (CV) and EIS in 0.1 M PBS (pH 7.4) containing 5 mM [Fe(CN)_6_]^4−/3-^. Voltammograms were recorded from −0.5 to 0.8 V at a 50 mV/s scan rate. The bare gold (Au), cystamine modified layer (Au/Cys), UEA-modified electrodes (Au/Cys/UEA), and BSA-treated probe (Au/Cys/UEA/BSA) were used for sensor characterization. EIS measurements were collected using a 2-electrode setup (RE and CE tied together) with an open-circuit voltage applied from 100 kHz to 0.1 Hz and an ac amplitude of 5 mV. Data from Nyquist plots were fitted with ZSimpWin software. A Randles equivalent circuit was used to extract the fitted charge transfer resistance, *R*_ct_.

### Human milk assay

The 2′FL sensors were tested against a range of 2′FL concentrations by serially diluting purified 2′FL samples. The sensor was incubated with various concentrations (500 to 4500 nM) in 0.1 M PBS (pH 7.4) for 30 min at room temperature and measured using EIS with 5 mM [Fe(CN)_6_]^4−/3-^ in 0.1 M PBS. Experiments were replicated (*n* ≥ 3) on independent sensor surfaces. As shown in Fig. 1, for the clinical samples, human milk was diluted, and 25 μL was drop-cast on the working electrode. After a 30-min incubation, EIS was performed in the same manner described previously. The change in *R*_ct_ (∆*R*_ct_) was read out and used to quantify the amount of 2′FL in the samples.

### Milk collection and storage

Milk samples stem from a previously reported study described by Plows et al. (2021) analyzed for HMO concentrations by HPLC, including 2’FL [32]. In brief, human milk was collected at least 1.5 h after the previous feeding and after the mother had fasted for at least 1 h. Participants provided a single full breast expression from the right breast in our clinical unit using an electric breast pump, ensuring the collection of fore-, mid-, and hind-milk. Human milk was mixed, aliquoted (into 10× 500-μL tubes, and the remainder into 5-mL tubes), and stored at −80 °C. 2′FL results quantified by HPLC were blinded to the researchers performing sensor studies and unblinded after sensor results became available. Institutional review boards at the University of Southern California and Children’s Hospital Los Angeles approved the study, where the human milk samples were collected as part of a larger study.

### Statistical analysis

All data was from a minimum of three independent experiments, and error bars represent one standard deviation. Statistical analysis was performed with Origin 9.0. The limit of detection (LOD) was calculated using LOD = 3.3 × standard deviation (SD) of the regression line/slope (95% confidence level) [33]. Linear regression with Deming’s method was used to interpret the comparison data [34].

## Results and discussion

### Sensor surface characterization

**Fig. 2:**
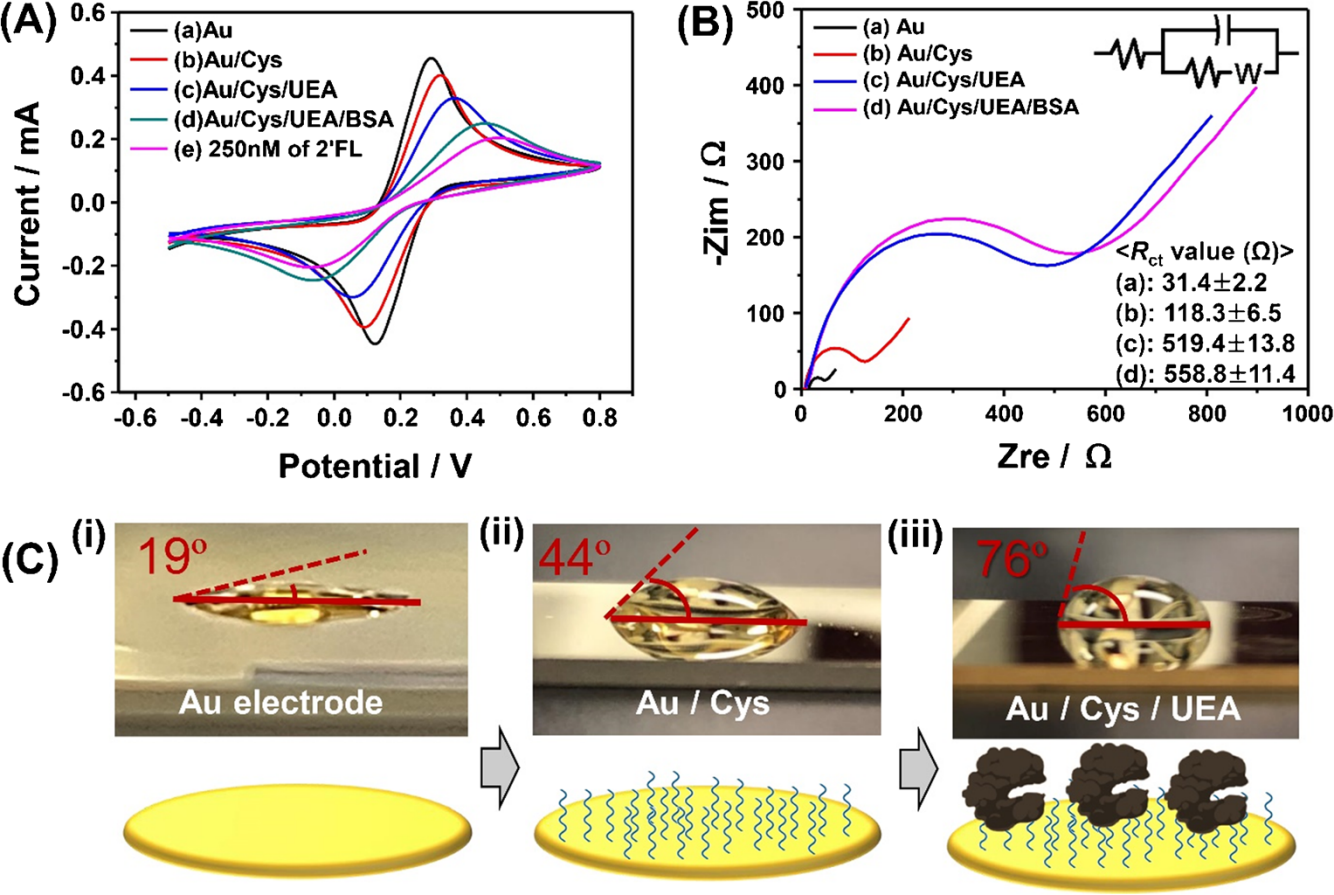
Stepwise electrode fabrication study. (A) Voltammograms of 2′FL sensor fabrication in 5 mM Fe(CN)_6_^4−/3−^. (B) Nyquist plots of 2′FL sensor fabrication steps. (C) Photographs of water droplets to quantify contact angle. (i) Bare Au substrates after acid treatment, (ii) Cys modified layer, and (iii) UEA coated with a Cys monolayer

The sensors were constructed by attaching UEA to the surface with amine chemistry. Voltammograms and spectra were recorded using CV and EIS, respectively, to characterize the stepwise sensor fabrication. CV is a valuable technique to determine the conductivity of the immobilized materials on the electrode surface using the current response of the mediator [35, 36]. Figure 2A shows voltammograms recorded at each step in the process, with the following: (a) bare gold electrode (Au), (b) addition of cystamine (Au/Cys) to present an amine functional group, (c) addition of lectin affinity reagent (Au/Cys/UEA), (d) blocking the surface with BSA (Au/Cys/UEA/BSA), and (e) the detection of the target HMO (Au/Cys/UEA/BSA/2′FL). Well-defined reversible redox peaks of ferri/ferrocyanide for the bare Au layer were observed at 128 and 284 mV. The peak current was reduced with the subsequent addition of cystamine (Au/Cys), inhibiting electron transfer, which indicated that the electrode conductivity was decreased by the covalent bond formed between Cys and Au (Au-S bonding). UEA and BSA immobilization further reduced the current response due to the accumulation of non-conductive proteins on the surface. Lastly, adding 250 nM of 2′FL to the sensor slightly decreased the peak current, indicating binding.

Next, EIS spectra were recorded to study the surface properties of the sensor, as quantified by the charge transfer resistance, *R*_ct_, since this is the most sensitive parameter to characterize what is happening on the electrode surface [35, 37]. To obtain the *R*_ct_ values, the Nyquist plot was fitted with a Randles equivalent circuit model (inset of Fig. 2B). As shown in Fig. 2B, *R*_ct_ increased with the stepwise immobilization due to the adsorbed non-conductive substances on the sensor surface. Because of the high electron transfer rate, the bare Au sensor had the lowest *R*_ct_ (31.4 ± 2.2 Ω). After adding the self-assembling Cys, *R*_ct_ increased to 118.3 ± 6.5 Ω due to increased steric hindrance and further increased when UEA was immobilized (519.4 ± 13.8 Ω), and the surface was blocked with BSA (558.8 ± 11.4 Ω) to mitigate non-specific binding. These data indicate that the capture probe was successfully attached to the sensor surface. These values are consistent with the CV responses. Finally, Fig. 2C shows contact angle measurements made at different stages of the modification process. The piranha-cleaned Au surfaces are hydrophilic (Fig. 2C, i), where acid treatments are widely used to eliminate organic contaminants and generate a hydrophilic surface. The self-assembled Cys monolayer (Fig. 2C, ii) had an increased contact angle (44°) because of the successful monolayer formation. Figure 2C, iii, shows the contact angle (76°) after adding UEA, indicating that the surface is now relatively hydrophobic. These results collectively indicate that the sensor is well-formed and demonstrates that it is responsive to the target HMO.

### Optimization

To maximize the response towards 2′FL, the assay parameters were systematically optimized. Specifically, the UEA immobilization time, 2′FL incubation time, and assay pH were studied. EIS was used to assess the effect of each parameter by quantifying the change in *R*_ct_ (Δ*R*_ct_). As shown in Electronic Supplementary Material Fig. S1A, EIS was performed at various times during UEA immobilization on the Au/Cys layer. For the UEA incubation time, Δ*R*_ct_ monotonically increased from 3 to 10 h and then plateaued. Thus, 10 h was chosen as the optimal condition. The target molecule incubation time, the critical factor in deciding the assay time, was investigated from 5 to 45 min (see Electronic Supplementary Material Fig. S1B) similarly. Δ*R*_ct_ increased linearly from 5 to 20 min and leveled off after 30 min, likely due to saturating all the available binding sites. Finally, the effect of measurement buffer pH was studied, ranging from 5.5 to 8.0, where pH 7.4 showed the maximum response (see Electronic Supplementary Material Fig. S1C). These optimum assay conditions were used for all subsequent experiments.

### Interference

**Fig. 3:**
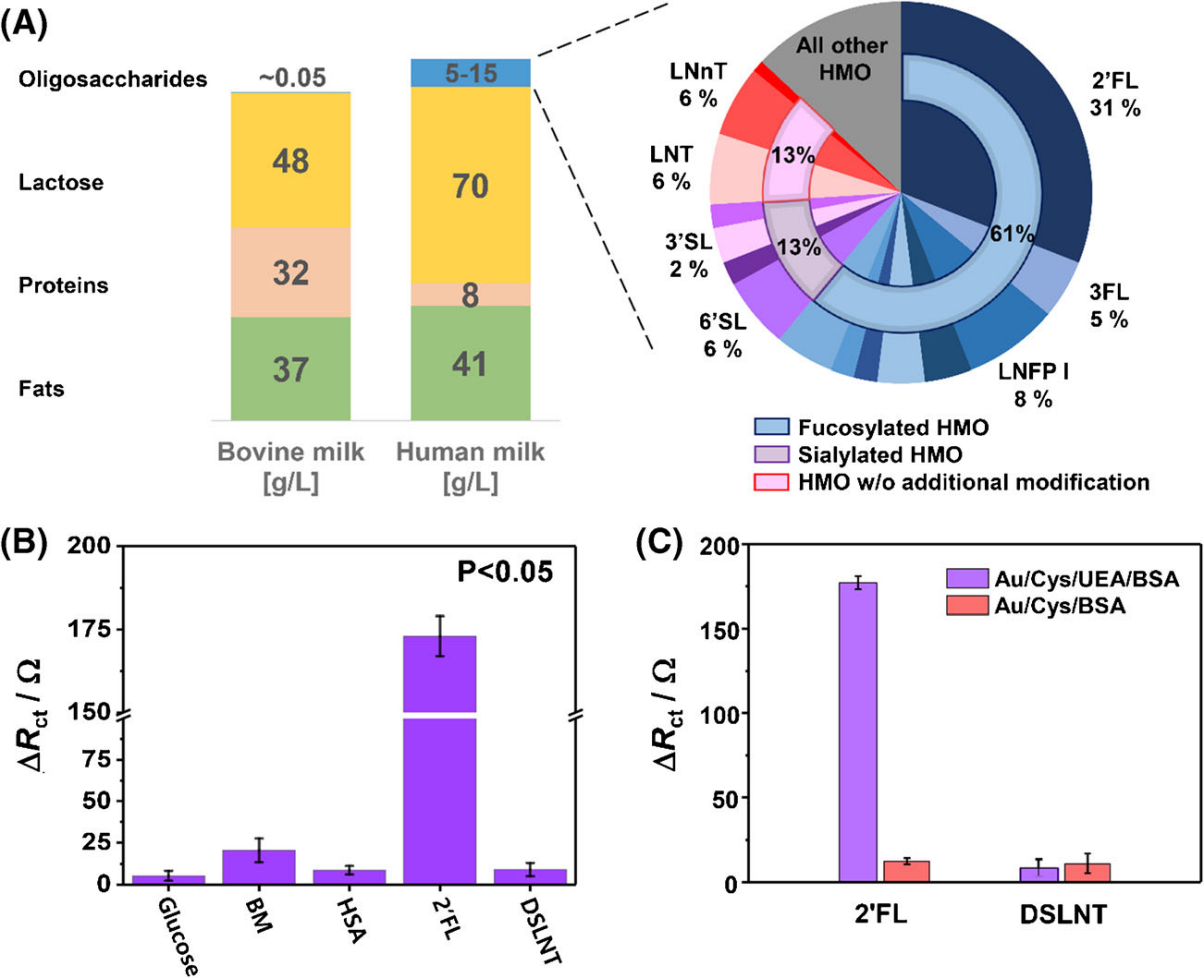
Interference study. (A) Bovine and human milk composition (left). Pull-out pie chart showing the composition of the most abundant HMOs. (B) Comparison of biosensor response to 1.0 μM 2FL and interfering species: glucose (0.5 mg/mL), 10-fold diluted bovine milk, HSA (1.0 mg/mL), and DSLNT (1.0 μM). (C) Selectivity of the sensor against 1.0 μM 2′FL (with ɑ-1,2 linkage) and DSLNT (without ɑ-1,2 linkage) in the absence (red) or presence (purple) of the affinity lectin, UEA. All measurements were performed in triplicate, where error bars indicate ±1σ

Human milk is a complex sample with an abundance of proteins, lipids, and sugars. The target HMO, 2′FL, is present at 2–5 g/L, whereas there is 5–15 g/L of total HMO [8], along with proteins at 8 g/L, fat at 41 g/L, 0.25 mg/mL of glucose [38], etc., as shown in Fig. 3A. HMOs are diverse with over 60% being fucosylated (*e.g.*, 2′FL, 3-fucosyllactose (3FL), lacto-*N*-fucopentaose I (LNFP I)), 13% sialylated (*e.g.*, 3′-sialyllactose (3′SL), 6′-sialyllactose (6′SL)), and some without any modifications (*e.g.*, lacto-*N*-tetraose (LNT), lacto-*N*-neotetraose (LNnT)), etc. Notably, non-secretors have significantly lower or absent 2′FL and LNFP 1 due to the inactive FUT2 enzyme [39].

To study the effect of these off-target molecules’ impact on the assay performance, we measured the sensor response in the presence of glucose (0.5 mg/mL; 2.78 mM), 10× diluted bovine milk (3.3 g/L of proteins, 0.1–0.2 g/L of bovine milk oligosaccharide [40, 41]), HSA (1.0 mg/mL; 15.1 μM), disialyllacto-N-tetraose (DSLNT; 1.0 μM), and 2′FL (1.0 μM). As shown in Fig. 3B, the sensor responses were negligible to these interfering species (*P* value <0.05), and the sensor showed a strong response to 2′FL. This experiment has two essential negative controls: bovine milk, which contains HMO analogs [42], and DSLNT, another HMO that, unlike 2′FL, is not α-1-2-fucosylated, but carries sialic acid instead. Critically, the sensor showed minimal response to these off-target glycans and robust signal to 2′FL. To further validate this and prove that the interaction is specific, we measured the sensor response to 2′FL and DLSNT at 1.0 μM on sensors with and without the affinity lectin, UEA. As shown in Fig. 3C, in the absence of UEA (red bars), the sensor showed a similar response to both 2′FL (12.3 ± 1.6 Ω) and DSLNT (11.0 ± 5.8 Ω). Meanwhile, with the affinity lectin immobilized, the sensor exhibited a strong signal for 2′FL (177.1 ± 3.9 Ω) and very little signal for DSLNT (8.5 ± 4.6 Ω). These results indicate that the sensor can selectively detect 2′FL by recognizing the ɑ-1,2 linkage of 2′FL and is not sensitive to off-target molecules.

### Detection of 2′FL

**Fig. 4:**
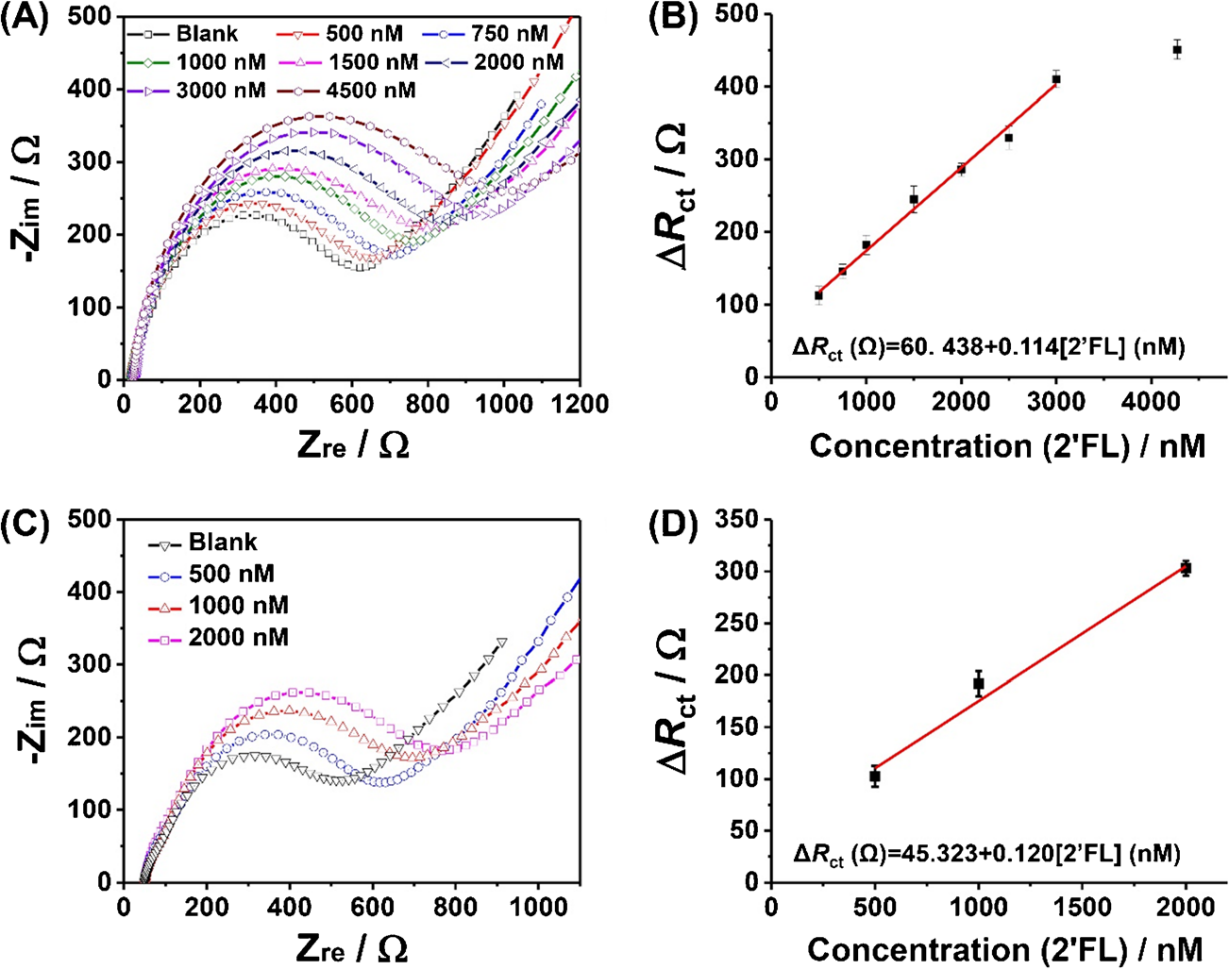
2′FL detection in buffer and human milk. (A, C) Nyquist plots of the sensor exposed to different concentrations of 2′FL in buffer and diluted, non-secretor human milk, respectively. (B,D) Extracted charge transfer resistance change (Δ*R*_ct_) vs. 2′FL concentration for respective Nyquist plots. All measurements were performed in triplicate, where error bars indicate ±1σ

The sensor’s analytical performance was investigated using EIS in 0.1 M PBS (pH 7.4) containing ferri-/ferro-cyanide. For each measurement, a stock sample containing 2′FL in PBS was serially diluted and assayed by placing 25 μL on the sensor and incubating for 30 min. As shown in Fig. 4A and B, the Nyquist plots showed a linear proportionality between *R*_ct_ and the 2′FL concentration from 0.5 to 3.0 μM. The linear regression equation for the plot in the standard sample is Δ*R*_ct_ = 60.438(±7.035) + 0.114(±0.005) (2′FL in nM) with a correlation coefficient of 0.991. The limit of detection (LOD) is 330.7 nM (161.5 ng/mL), which is superior to the previously reported methods [23, 28, 43, 44]. To define a secretor and non-secretor, a cutoff of 410 μM was chosen based on previous reports [45]. Therefore, the developed 2′FL sensor can be used as a milk analyzer to distinguish secretor/non-secretor status using 1000-fold diluted milk samples. As shown in Fig. 4C and D, the developed sensor was evaluated with 2′FL spiked (0.5, 1.0, and 2.0 μM) in human milk samples (non-secretor) diluted 1000-fold. Despite the more complex milk matrix, the measured *R*_ct_ values were still proportional to the 2′FL concentration. The linear regression equation is Δ*R*_ct_ = 45.323(±8.035) + 0.120(±0.01) (2′FL in nM) with a correlation coefficient of 0.98. Compared to the PBS samples, the milk response was slightly increased, which is likely due to the complexity of the milk matrix and the existence of unknown interfering species adsorbing on the sensor.

### Reproducibility and long-term storage stability

**Fig. 5:**
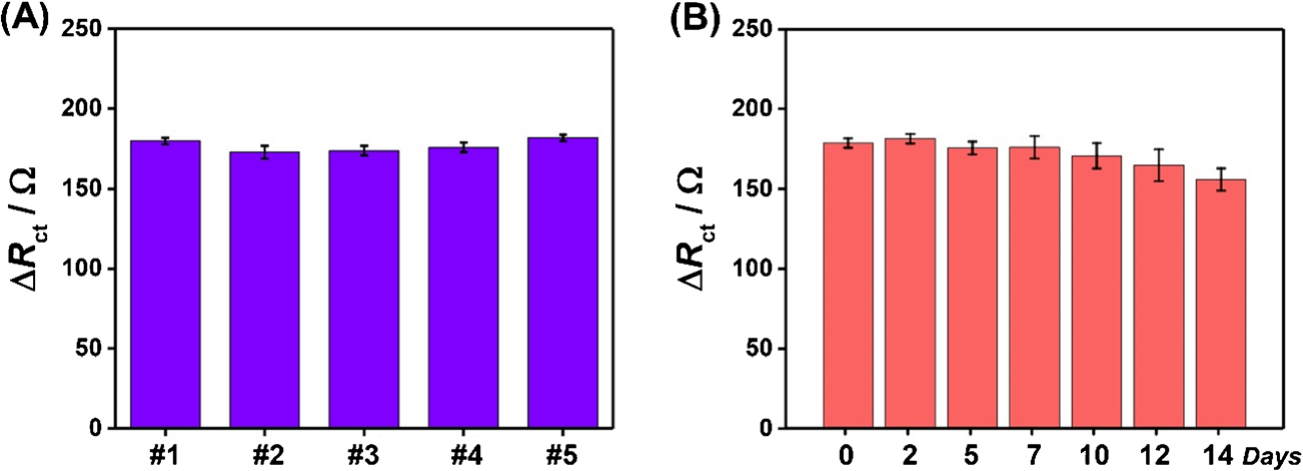
Reproducibility and long-term storage. (A) Measured ∆*R*_ct_ of 1.0 μM 2′FL on independent sensors (*n* = 5). (B) Measured ∆*R*_ct_ over time to show long-term stability. All measurements were performed in triplicate, where error bars indicate ±1σ

Sensor fabrication consistency is essential to achieve reproducibility [46], which can be achieved by improving the uniformity of the self-assembled monolayers (SAM) and the covalently attached receptors on the sensor surface. In particular, the sensor surface cleanness and reagent contamination significantly affect the SAM uniformity and cross-linked receptors [47]. Therefore, the substrates were thoroughly washed with chemical and electrochemical methods prior to sensor fabrication using freshly prepared reagents. EIS was performed on five independent sensors produced from the same batch of substrates, and the coefficient of variation (CV) was calculated to assess the consistency. Figure 5A shows the measured responses to 1.0 μM of 2′FL where the CV was less than 4.7% for 15 measurements, indicating the sensor fabrication and response were highly reproducible. Next, the sensor’s long-term storage stability was evaluated by measuring the sensor response over a 2-week period. Sensors were prepared and then stored in a refrigerator at 4 °C and covered with parafilm between measurements. The sensors were periodically removed, incubated with 1.0 μM of 2′FL, measured, and washed. As shown in Fig. 5B, the sensors retained more than 95% of their initial day 0 response for up to 10 days, 92% at 12 days, and decreased slightly to 88% after 14 days. These results demonstrate that the Au/Cys/UEA/BSA functionalized sensor has high reproducibility and maintains stability over a long period. For binary detection (secretor vs. non-secretor), the sensors are likely stable for an even longer time, although that was not explored in this work.

### Clinical validation

**Fig. 6:**
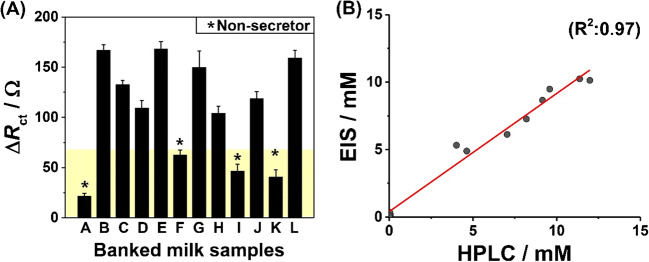
Clinical measurements. (A) Electrochemical signal (Δ*R*_ct_ values) of banked human milk samples (*n* = 12) (non-secretor status marked with *). (B) Comparison of EIS and HPLC data. All measurements were performed in triplicate, where error bars indicate ±1σ

To verify the clinical applicability, donor human milk samples were quantified using the developed electrochemical sensors. Banked human milk samples were obtained from the Mother-Milk-Infant Center of Research Excellence (MOMI CORE) at the University of California San Diego and prepared as described above. Samples (*n* = 12) were provided blinded and tested using the proposed assay. As shown in Fig. 6A, 4 of the 12 samples (A, F, I, and K) had signals below the cutoff threshold and were identified as non-secretor milk, while the others were called secretors. Those identified as secretors (B, C, D, E, G, H, J, and L) were then 10× further diluted and re-run to bring their signal within the sensor’s dynamic range for quantification. After unblinding the samples, all agreed with the HPLC data (100% positive percent agreement and 100% negative percent agreement). While the sample size is relatively small, the concentration range is representative and highly encouraging. Furthermore, the error bars on the replicate samples are small, indicating little variability in the assay. The EIS results were compared against the gold standard measurement technique (HPLC) run on the same samples (Electronic Supplementary Material Table S1). The agreement between the methods was evaluated using a Deming method comparison test [34]. As shown in Fig. 6B, there was no significant difference between the two analytical methods as the EIS results exhibit a strong correlation with the HPLC data (*R*^2^ = 0.97). The data were also evaluated using the paired *t*-test, and the calculated *t* value (1.42) was less than the critical *t* value (2.365) at the 95% confidence level (*n* = 8). It is worth pointing out that HPLC is selective against just 2′FL, whereas this test measures all HMOs with an α-1,2 linkage, of which 2′FL is the most abundant. These data demonstrate that the proposed sensor can accurately quantify 2′FL and be used to differentiate secretor vs. non-secretors at the bedside.

### Comparison

Over the last several decades, various analytical methods to quantify 2′FL have been developed, such as gel filtration with paper chromatography, HPLC, hydrophilic interaction liquid chromatography with fluorescence detection (HILIC-FLD), high-performance anion**-**exchange chromatography (HPAEC), and a whole-cell biosensor with fluorescence detection. Their performance is summarized and compared in Table 1. The developed impedimetric 2′FL sensor has the lowest limit of detection (LOD) and the fastest assay time (35 min). Notably, the proposed sensor requires no preprocessing steps (such as centrifugation), enabling it to be used for point-of-care testing. In addition, this work is the most cost-effective (<$3/test) (see Electronic Supplementary Material Table S2 for a cost breakdown). While electrochemical techniques have an affordable instrumental cost considering the device portability (<$5000), HPLC and multiplexed HPLC methods require a specific detector and column for each purpose and high instrument cost (~$27,500) [48]. The reader for the developed assay can be miniaturized [49–51], enabling screening human milk on-site and hence used as a bedside milk analyzer.

**Table 1:** Analytical performance of 2′FL detection methods

Method	Probe	Sample matrix	Sample Prep	LOD (μM)	Assay time (min)	Assay cost	Portable?
Gel filtration and paper chromatography [52]	Sephadex 25	Human milk	Centrifuged		–	$$	No
HPLC with refractive index detection [23]	Amide column	Infant formula	Centrifuged	200	120	$$$	No
HILIC-FLD [28]	Glycan and amide column	Infant formula in DMSO	Centrifuged	8.9	240	$$$	No
Whole-cell biosensor with fluorescence detection [43]	*E. coli* and enzyme reaction	Recombinant	GFP tagged 2′FL	20	360	$$	No
HPAEC [44]	Amide column	Human milk, urine, and plasma	Centrifuged	0.88	60	$$$	No
EIS (this work)	UEA	Human milk	Diluted	0.33	35	$	Yes

## Conclusion

This work demonstrated a label-free, rapid, simple, low-cost analysis platform to differentiate secretor and non-secretor status in human milk. Selective detection is accomplished using a lectin affinity reagent towards HMOs with α1-2-fucosylation, such as the highly abundant 2′-fucosyllactose. The sensor is read out using an electrochemical impedimetric assay where 2′FL selectively binds to UEA and decreases the charge transfer resistance. This scheme was optimized and then validated using PBS and non-secretor donor milk spiked with 2′FL. The assay specificity against off-target HMOs and potential interferers was verified, with little to no cross-reactivity observed. The reproducibility and stability were demonstrated over a 2-week timeframe with a low coefficient of variation. This set the stage for clinical validation where donor human milk was screened for secretor status. The results were in perfect agreement with HPLC despite the ~4× shorter assay time and significantly lower cost. A fast HMO assay with a low sample volume (<25 μL) enables on-site, bedside human milk monitoring throughout lactation and could result in better infant health outcomes. This test could also be used for donor human milk screening to decide on the use of secretor vs. non-secretor milk once research studies confirm the benefits of one over the other depending on a given situation and context. While the proposed assay offers considerable promise to manage variation in human milk composition due to its simplicity, user-friendly, and fast response time, it requires further clinical validation with a larger sample size. Future work will expand this strategy to other HMOs and human milk bioactives for a more holistic milk composition assessment. This assay provides a new perspective in milk research and lays the foundation for a POC bedside milk analyzer for clinical purposes and a milk screening tool.

## Supplementary information

ESM 1 (DOCX 267 kb)
