# Supplementary material for: Point-of-care human milk testing for maternal secretor status
Source: Anal Bioanal Chem. 2021 Nov 5;414(10):3187–96. doi: 10.1007/s00216-021-03697-7 (PMC8956550; doi:10.1007/s00216-021-03697-7)
Supplement: Supplementary file 1 — (DOCX 267 kb) [file 216_2021_3697_MOESM1_ESM.docx]

**Supporting Information**

Point-of-care human milk testing for maternal secretor status

**Author names and affiliation:** Saeromi Chung ^a^, Lars Bode ^b^, Drew A. Hall ^a,c,*^

^a^ Department of Electrical and Computer Engineering, University of California – San Diego, La Jolla, CA 92093, USA

^b^ Department of Pediatrics and Mother-Milk-Infant Center of Research Excellence (MOMI CORE), University of California – San Diego, La Jolla, CA 92093, USA

^c^ Department of Bioengineering*,* University of California – San Diego, La Jolla, CA 92093, USA

^*^ **Corresponding author:** [drewhall@ucsd.edu](mailto:drewhall@ucsd.edu)


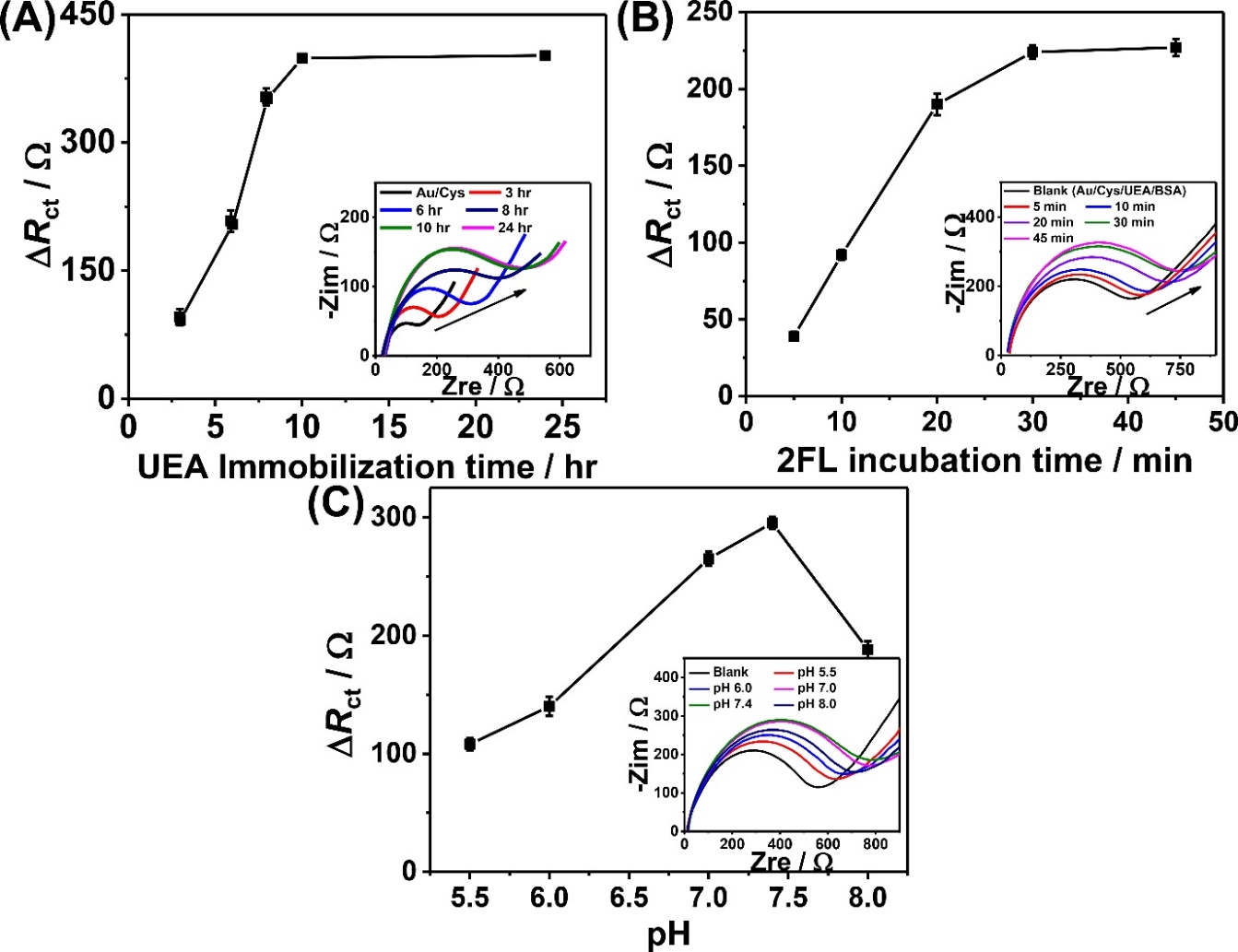


**Figure S1**. **Optimization of experimental parameters.** **A**. UEA immobilization time, **B.** 2’FL incubation time with 1.5µM 2’FL, and **C**. pH with 2.0µM 2’FL in 0.1 M PBS containing 5 mM Fe(CN)_6_^3-/4-^.

**Table S1**. Comparison of concentration of banked samples (HPLC vs. EIS).

| **Sample ID** | **EIS (µM)** | **HPLC (µM)** | **Secretor/non-secretor?** | **Prediction** |
| --- | --- | --- | --- | --- |
| A | <330 | 5 | Non-secretor | Correct |
| B | 10129 | 11985 | Secretor | Correct |
| C | 7268 | 8197 | Secretor | Correct |
| D | 5323 | 4017 | Secretor | Correct |
| E | 10243 | 11378 | Secretor | Correct |
| F | <330 | 71 | Non-secretor | Correct |
| G | 8660 | 9159 | Secretor | Correct |
| H | 4894 | 4632 | Secretor | Correct |
| I | <330 | 34 | Non-secretor | Correct |
| J | 6126 | 7045 | Secretor | Correct |
| K | <330 | 5 | Non-secretor | Correct |
| L | 9484 | 9585 | Secretor | Correct |

**Table S2**. Cost analysis of proposed assay for 2’FL detection. All prices are from the published price on the vendor’s website in low quantity.

| **Item** | | **Unit Cost (USD)** | **Cost (USD)/batch (20×)** |
| --- | --- | --- | --- |
| Top 5 reagents | Cys | $15/10g | $0.15 |
|  | UEA | $44.40/1mg | $0.44 |
|  | Glutaraldehyde | $20/10mL | $0.10 |
|  | 10% BSA | $24.20/10mL | $0.01 |
|  | NH_4_OH | $40/1L | $0.10 |
| Electrode | Au electrode | $350/75 | $50.00 |
|  |  | **Cost per test** | **$2.50** |
|  |  |  |  |
